# Argon Laser Synechiolysis for Irido-Corneal Adhesion

**DOI:** 10.1155/2022/5289203

**Published:** 2022-04-06

**Authors:** Saeed Shokoohi-Rad, Samaneh Gholamhoseinpour-Omran, Hamed Hosseinikhah-Manshadi, Mohammad Baghi Yazdi

**Affiliations:** Eye Research Center, Mashhad University of Medical Sciences, Mashhad, Iran

## Abstract

**Purpose:**

To introduce a new method for treating irido-corneal adhesion (ICA) by using argon laser. *Case Presentation*. A 60-year-old woman with advanced glaucoma who is undergoing trabeculectomy. In follow-up visits, choroidal effusion and iridocorneal adhesion were seen in the examination. The massive choroidal effusion was treated with medical therapy. In further follow-up examination, ICA was repaired with argon laser.

**Result:**

Early laser treatment prior to surgical management is suggested in postoperative synechia.

**Conclusion:**

Early argon laser synechiolysis is a less-invasive procedure which prevents probable surgical complications such as local detachment of Descemet's membrane or bleeding in surgical synechiolysis.

## 1. Introduction

There has been great progress in uses of lasers for many forms of procedures to treating glaucoma. Some of the procedures include argon laser trabeculoplasty, selective laser trabeculoplasty, endoscopic cyclophotocoagulation, and laser-assisted deep sclerotomy. Other applications of laser procedures such as suture lysis, bleb reopening, and iris adhesion lysis for posterior and anterior synechiae exist in this treatment field [1].

In this study, we introduce one of the laser technique applications for irido-corneal adhesion (ICA) which is formed sometimes after trabeculectomy in patients with advanced glaucoma. In this case, we reported a patient with early successful treatment of irido-corneal adhesion with argon laser synechiolysis.

## 2. Case Presentation

A 60-year-old woman was referred to and eye hospital for continuing glaucoma management. The patient had history of angle closure glaucoma and cataract surgery of the right eye last year. In the initial evaluation, best corrected vision for both the right and left eyes was 6/10; relative afferent pupillary defect (RAPD) was 2+ for the right eye. The right eye was pseudophakic. Intraocular pressure (IOP) was 25 mmHg while using brimonidine tartrate 0.2%, dorzolamide 2%, timolol 0.5%, and latanoprost. IOP for the left eye was 10 mmHg without medication. The cup-to-disc ratio was recorded 100% for the right and 80% for the left eye. Both eyes had 270 degrees of peripheral anterior synechiae in gonioscopy.

The patient was a candidate for right eye trabeculectomy. Surgery was concluded by an experienced surgeon in glaucoma without complications during the operation. At follow-up visits, days 1 and 3 and a week after surgery, the eye was moderately injected, IOP was 12,9,11, respectively, and funduscopy was normal.

Two weeks after surgery, the patient returned again to the glaucoma clinic with complaints of decreased visual acuity. The intraocular pressure (IOP) was 17 for the right eye, and anterior chamber was flat symmetrically at peripheral and central, grade 2, and the bleb was low lying and focal (not diffuse). The diagnosis was malignant glaucoma, and soon, IZHV (irido zonular hyaloid vitrectomy) was done for patient. According to the probable mechanism of malignant glaucoma, most of aqueous misdirection fluids are accumulated and lobulated around the equator of lens; hence, the preferred intervention for this kind of patient is IZHV [2, 3]. Based on a review by Debrouwere et al., IZHV was a treatment of choice in management of malignant glaucoma in comparison with posterior YAG capsulotomy [4].

**Figure 1:**
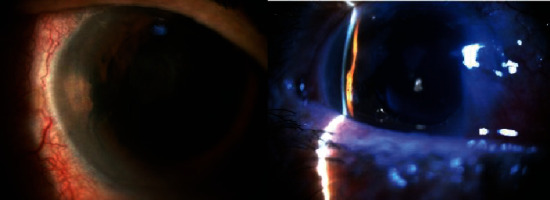
Slit lamp photograph of the right eye shows focal irido-corneal adhesion before argon laser synechiolysis.

In the next follow-up, nearly 10 days after IZHV, IOP was 7 mmHg, and on B-scan examination, nonmassive choroidal effusion was seen, since there was no indication of surgery for the patient; the patient was treated with tablet prednisolone 50 mg daily and followed closely. ICA (about one clock hour) was observed after healing of the choroidal effusion (Figure 1).

**Figure 2:**
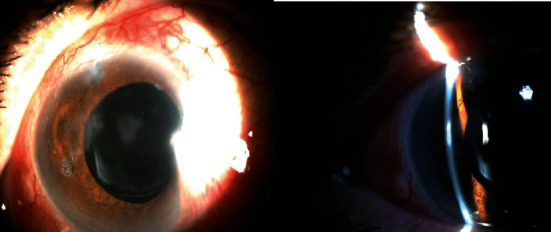
Slit lamp photograph of the same eye after argon laser synechiolysis showing symmetric deep anterior chamber.

Then, the patient undergone argon laser synechiolysis with spot size 100 micron, 0.1 second, and power 400 mW near the adhesion site. The anterior chamber was immediately deepened and formed in 360 degrees after argon laser synechiolysis. In subsequent follow-ups, the IOP was 11 mmHg, and anterior chamber was deep and formed, and there was no choroidal effusion (Figure 2).

Due to successful experience of this case, since ICA is a common complication in anterior segment surgeries (including DSAEK, PK, and Phaco) in the future, argon laser can be used as a supporting treatment for this complications.

## 3. Discussion

Laser treatment in glaucoma continues to evolve with attitude towards primary and earlier intervention. Initial usage of argon laser trabeculoplasty was demonstrated by Wise and Witter which was effective for lowering intraocular pressure (IOP) [5, 6].

Development of new laser techniques results to be an essential role for glaucoma treatment. Laser technique is a safe and effective therapeutic approach for posttrabeculectomy suture lysis, closing cyclodialysis cleft, enlarging miotic pupil, and removing peripheral anterior synechiae from the angle of cornea [7].

Peripheral anterior synechiae in early stage is removed by setting (400–800 mW, 0.1–0.2 second, 50–100 *μ*m spot size) as same as iridoplasty. It is essential to break down irido-corneal adhesin after trabeculectomy or penetrating keratoplasty in early stage. In our case, this procedure fourteen days after adhesion formation was successful, hence seems argon laser is not effective for chronic form of synechiae due to firmly adherent of iris to cornea [8].

Laser technique can reduce some surgical complication, for example, anterior synechiae removal by surgical dissection may have risks of local detachment of Descemet's membrane or bleeding. In this case, we support argon laser for lysis iridocorneal adhesion with nearly half of the mentioned power and spot size in early stage.

## Data Availability

The datasets used during the current study are available from the corresponding author on reasonable request.
